# No Support for White Matter Alterations in Adults With Dyslexia: A Fixel-Based Diffusion MRI Study

**DOI:** 10.1162/NOL.a.17

**Published:** 2025-09-24

**Authors:** Helena Verhelst, Robin Gerrits, Emma M. Karlsson

**Affiliations:** Department of Experimental Clinical and Health Psychology, Ghent University, Ghent, Belgium; Research Group Cognition and Plasticity, Max Planck Institute for Human Cognitive and Brain Sciences, Leipzig, Germany

**Keywords:** DWI, dyslexia, reading, tractography, white matter

## Abstract

Developmental dyslexia is a common learning disability marked by reading and spelling difficulties. While previous imaging studies aiming to elucidate the neurobiological mechanisms of this disorder have reported white matter alterations, they are inconsistent with regards to which specific tracts are implicated and in what way. These inconsistencies might partially stem from methodological limitations such as small sample sizes and the use of outdated diffusion models. To address these issues, we used fixel-based analyses, an advanced diffusion framework, to compare structural white matter organization between 35 adults with dyslexia and 34 controls across three levels of analysis (whole-brain, tract-specific, and tract-averaged). Contrary to expectations, none of the analyses yielded significant group differences. However, within the dyslexic group only, poorer word reading proficiency was linked to greater fiber density and cross-section of the bilateral inferior longitudinal fasciculus. Taken together with the existing literature, our results suggest that white matter structure might not be altered in (adulthood) dyslexia or might be idiosyncratically impacted to such an extent that group-average studies are unable to detect these changes. Future large-scale research and efforts to pool datasets across studies will prove essential for understanding the white matter correlates of dyslexia.

## INTRODUCTION

Developmental dyslexia is a common specific learning disability characterized by difficulties in reading and/or spelling. These impairments typically stem from a deficit in the phonological component of language and are not caused by neurological, psychiatric, sensory, intellectual or motivational issues (Lyon, 1999; Siegel, 2006). Although the disorder often is most apparent during school age, the difficulties usually persist into adulthood (Bruck, 1990, 1992; Shaywitz et al., 1999). While some symptoms may change over time and individuals often develop strategies to manage their challenges, core difficulties with reading, spelling, and language processing tend to remain (Moojen et al., 2020). Despite the absence of overt neural damage, dyslexia has been associated with different brain functioning (Norton et al., 2015). Altered brain activity has been reported in various studies, particularly in left hemisphere regions involved in oral language and reading, such as the occipitotemporal (visual word form area), temporoparietal, and inferior frontal gyrus (Kronbichler & Kronbichler, 2018; Li & Bi, 2022; Martin et al., 2016; Paulesu et al., 2014; Yan et al., 2021). Dyslexic individuals often show underactivation in these areas during reading tasks, alongside compensatory hyperactivation in right hemisphere regions and frontal areas. These neural patterns suggest a disruption in the typical left-lateralized reading network, contributing to difficulties in phonological processing, decoding, and fluent reading (Pugh et al., 2000; Richlan et al., 2009; Shaywitz & Shaywitz, 2008).

Since reading relies on a distributed network of brain regions (Price, 2012; Rueckl et al., 2015), one could hypothesize that the physical connections between these areas may be compromised in dyslexia, whether as a cause, consequence, or risk factor (Bishop, 2013). Although several neuroimaging studies have used diffusion-weighted imaging (DWI) to investigate white matter properties in dyslexia, both in a whole-brain voxelwise manner, as well as in specific, preselected tracts of interest, the white matter correlates of this reading disorder have remained elusive (Ramus et al., 2018). An early meta-analysis supported that white matter microstructure is less developed in left temporoparietal and frontal parts of the brain in dyslexia according to voxelwise diffusion tensor imaging (DTI) studies (Vandermosten, Boets, Wouters, et al., 2012). However, a subsequent activation likelihood estimation meta-analysis that also included later studies found no reliable clusters of white matter structures that differed between people with and without dyslexia (Moreau, Stonyer, et al., 2018). While voxel-based approaches offer the advantage of comprehensive whole-brain coverage, enabling the identification of highly localized effects, they may overlook group differences that are spatially diffuse across individuals within specific tracts. Moreover, the large number of statistical tests necessities more stringent multiple comparison testing compared to analyzing a subset of white matter tracts.

Research examining specific white matter tracts of interest mostly based their selection on the well-established dual route model of reading, which proposes that reading relies on two distinct mechanisms (Coltheart et al., 1993). The sub-lexical pathway, also known as the phonological route, uses stored spelling-to-sound rules to convert graphemic input into a phonological output representation, which can then be used to access its meaning. Although this mechanism is a relatively slow way of processing written language, it does provide a means to read unfamiliar words and meaningless strings. By contrast, the lexical or orthographical pathway directly maps orthographic units such as morphemes or words onto their semantic representation, allowing rapid lexico-semantic retrieval. These two pathways are subserved by distinct neural substrates (Jobard et al., 2003; Joubert et al., 2004; Roux et al., 2012; Turker & Hartwigsen, 2021). The sub-lexical pathway comprises dorsal white matter bundles such as the arcuate fasciculus (AF) and superior longitudinal fasciculus (SLF). The AF follows a distinctive curved trajectory around the Sylvian fissure to link posterior temporal and inferior frontal regions, while the SLF extends longitudinally along the dorsal aspect of the brain, linking temporo-parietal and frontal regions. In contrast, the lexical pathway is underpinned by ventral white matter tracts, including the inferior longitudinal fasciculus (ILF), which connects the occipital lobe with the anterior temporal lobe, and the inferior fronto-occipital fasciculus (IFOF), which connects the prefrontal cortex with parietal, posterior temporal and occipital regions (Vandermosten, Boets, Wouters, et al., 2012; Yablonski et al., 2019).

The tract most often considered by previous studies on dyslexia is the AF (Ramus et al., 2018), likely because of its historical link with language functioning (Dick & Tremblay, 2012). Although the majority of studies observed lower values of fractional anisotropy (FA), a metric of white matter microstructure, in poor readers (Farah et al., 2022; Vanderauwera et al., 2017; Vandermosten, Boets, Poelmans, et al., 2012; Zhao et al., 2016), some failed to find significant group differences (Meisler & Gabrieli, 2022; Moreau, Wilson, et al., 2018). For the SLF, several studies reported increased anisotropy in the right hemisphere of poor readers compared to typical readers (Banfi et al., 2019; Sihvonen et al., 2021; Zhao et al., 2016), while others noted lower FA on the left (Farah et al., 2022) or in both hemispheres (Slaby et al., 2023). Results in the literature regarding the ventral tracts are equally contradictory. For example, group differences in the IFOF observed by some studies (e.g., Zhao et al., 2016) are absent in others (Banfi et al., 2019; Vandermosten, Boets, Poelmans, et al., 2012). Similarly, depending on the study, the FA of the ILF was been reported to be reduced (Farah et al., 2022), higher (Banfi et al., 2019), or unaffected in dyslexics (Zhao et al., 2016). In sum, several individual studies that adopted the tracts-of-interest approach have observed changes in white matter properties in dyslexia, but they vary in which specific tracts are implicated and the direction of the effect (for a systematic review, see Ramus et al., 2018).

The inconsistencies in the literature on the white matter correlates of dyslexia might partially be due to the challenges posed by limited sample sizes (Ramus et al., 2018). Small-scale studies frequently result in spurious significant findings and inflated effect sizes, increasing their likelihood of being published compared to null results. The sample size issue is sometimes compounded by absent corrections for multiple testing. For instance, the meta-analysis of Moreau and colleagues suggested that significant findings of some of the earlier voxel-wise studies might have resulted from a lack of an appropriate correction for the many comparisons such an approach entails (Moreau, Stonyer, et al., 2018). Apart from small sample sizes and inadequate corrections for simultaneous testing, the heterogeneity in the results of previous white matter studies may also stem from differences in methodological decisions between studies, such as inclusion criteria (e.g., the definition of *dyslexia*), the level of analysis (voxelwise vs. tract-of-interest approach), and tract quantification methods (Ramus et al., 2018). A major pitfall in white matter research is the frequent use of the limiting and outdated DTI model. The issue of DTI is that it assumes diffusion occurs in a single direction per voxel, oversimplifying the complex structure of white matter tracts. In areas with crossing, branching, or densely packed fibers, this can result in inaccurate measurements and poor reconstruction of white matter pathways (Jones et al., 2013).

Newer and more advanced models and frameworks have been developed to overcome the limitations of the DTI model (Jeurissen et al., 2013; Jones et al., 2013; Tournier et al., 2011). For example, fixel-based analyses (FBA) enable the quantification of micro- and macrostructural properties of white matter of multiple fiber populations within a voxel (called *fixel*; Raffelt et al., 2017). Within the FBA framework, statistical inferences can be performed on these fixels using the fixel-derived metrics fiber density (FD), fiber bundle cross-section (FC), and the combined measure fiber density and cross-section (FDC). FD reflects the density of fibers oriented along a specific direction within a voxel, FC estimates the cross-sectional area of a fiber bundle, and FDC integrates both FD and FC to provide a comprehensive measure that captures changes in both fiber density and bundle area (see Dhollander et al., 2021, for a comprehensive review on FBA and its metrics). Since FDC is proportional to axonal volume, it serves as an indirect marker of the biophysical properties of white matter tracts, including action potential conduction velocity, which is influenced by factors such as axonal diameter and myelination (Raffelt et al., 2017; Waxman, 1980). A recent study by Meisler and Gabrieli (2022) used the FBA framework to examine whether reading abilities are related to white matter structure in a large dataset of 983 children and adolescents aged 6–18. They found positive correlations between FDC and reading skills throughout the brain, especially in left temporoparietal and cerebellar white matter. However, no significant group differences were detected when they compared subgroups of poor and typical readers.

**Figure 1.**
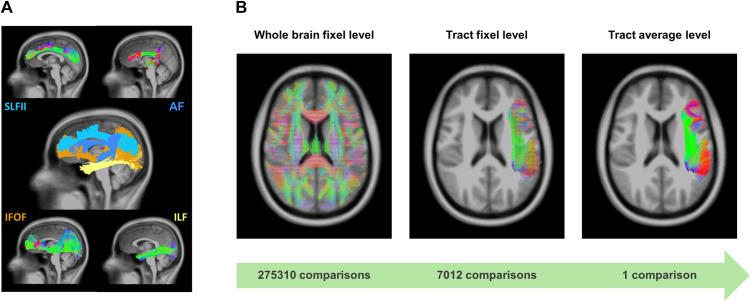
White matter tracts-of-interest and definition of the levels of analysis. (A) Tracts of interest. (B) The three levels of analysis. SLF = superior longitudinal fasciculus, AF = arcuate fasciculus, IFOF = fronto-occipital fasciculus, ILF = inferior longitudinal fasciculus.

Given the shortcomings and contradictory results of earlier DWI research on dyslexia, the current study aimed to revisit whether white matter is altered in adults diagnosed with dyslexia. Specifically, we addressed previous methodological challenges by (1) using the advanced diffusion framework of FBA, (2) applying this framework on three different levels of analysis, and (3) appropriately correcting for multiple testing. First, we perform a standard whole-brain fixel-based group comparison of FDC since this approach comes with the greatest spatial specificity. Next, we performed fixel-based group comparisons of FDC using inclusion masks of two dorsal route tracts (AF and SLF) and two ventral route tracts (IFOF and ILF). By constraining the comparisons to only those fixels that belong to a priori defined tracts, we reduced the number of comparisons considerably and thus increased the statistical power. Finally, we averaged the FDC metrics over the tracts, further limiting the number of comparisons by having only one FDC value per tract. This approach also considers the possibility that alterations within a white matter tract might be expressed in different segments across individual dyslexics. If this is the case, it can show up in group differences in the average FDC of the tract. To examine whether there are associations between white matter and reading proficiency in dyslexia, we performed correlations between FDC and single word reading performance in the dyslexia subgroup. Here, we also carried out the analyses on three levels. A visual representation of the three analysis levels is provided in Figure 1.

## MATERIALS AND METHODS

### Participants

Thirty-five adults with a diagnosis of dyslexia and 35 individuals without any diagnosis of language or reading impairment were recruited for this study via social media, word of mouth, and a student participation platform. Inclusion criteria were Dutch as a first language, age between 18 and 40, and right-handedness. All participants had normal or corrected-to-normal vision and reported no history of brain injury or neurological disease. All participants in the dyslexia group received a diagnosis of dyslexia from a trained professional, either while they were at school or in the context of receiving disability services at university/college. During the analysis phase, one control participant was excluded because of poor magnetic resonance imaging (MRI) data quality. Specifically, their mean FDC values deviated from the group average by more than 2.5 times the standard deviation across all tracts of interest. As a result, the final analyses were conducted on a total sample of 69 participants (35 dyslexia + 34 controls). The groups did not differ significantly in terms of age, sex distribution, intracranial volume, or years of formal education (all *p*s > 0.05; see Table 1). The study was approved by the medical ethical committee of Ghent University (approval number BC-09822) and a written informed consent was obtained from each participant.

### Behavioral Measures

To confirm that dyslexia-related language difficulties persisted into adulthood, participants were given three language proficiency tests sufficient for correctly diagnosing dyslexia in adults: word reading, word spelling, and phonological awareness (Tops et al., 2012). In the Word Reading Test for Students (Tops et al., 2019), participants are required to read out loud as many words as possible within a minute. Word spelling and phonological awareness was assessed using the GL&SCHR, a reading and spelling test battery normed for Flemish-Dutch young adults. The word spelling subtest of the GL&SCHR involved participants writing down 30 exception words read out to them (Depessemier & Andries, 2009). In line with the findings of Tops et al. (2012), a weighted score was computed that considered both accuracy and confidence in spelling. Phonological awareness was evaluated using the Spoonerisms subtest from the GL&SCHR, where participants had to switch the first letters of two auditorily presented words, such as transforming “Harry Potter” into “Parry Hotter.” Once again, a weighted score was calculated that factored in accuracy and reaction time.

### MRI Data Acquisition

MRI data were acquired using a 3T Siemens PRISMA scanner with a 64-channel head coil. Diffusion MRI (dMRI) data was acquired for 11 and 60 non-collinear gradient directions respectively using b-values of 0 and 3,000 s/mm^2^. Additionally, an extra pair of b = 0 images was acquired using opposite phase encodings, to allow for correction of susceptibility induced EPI distortions. Other acquisition parameters are as follows: 2 mm isotropic voxel size, 75 axial slices, FoV = 240 mm, TR/TE = 3,200 ms/77 ms.

In addition to the dMRI, a high-resolution T1-weighted image was collected with the following parameters: 1 mm isotropic voxel size, 176 sagittal slices, FoV = 256 mm, TR/TE = 2,250 ms/4.18 ms. The T1-weighted volume was used for creating volumes of interest for the automated tractography (see below) and to estimate intracranial volume using FreeSurfer (Fischl, 2012).

### Fixel-Based Analyses

The FBA was performed using MRtrix3 (Tournier et al., 2019), following the recommended protocols outlined by Dhollander et al. (2021) and Raffelt et al. (2015, 2017).

Preprocessing of the dMRI data included denoising (Veraart et al., 2016), removal of Gibbs ringing artifacts (Kellner et al., 2016), and corrections for motion, eddy currents, and EPI distortions (Andersson et al., 2003, 2016; Andersson & Sotiropoulos, 2016). Subsequently, the preprocessed data were upsampled to a voxel size of 1.5 mm isotropic.

White matter fiber orientation distributions (FODs) were estimated by a single-shell 3-tissue constrained spherical deconvolution approach (SS3T-CSD) from MRtrix3Tissue (https://3Tissue.github.io), a fork of MRtrix3 (Tournier et al., 2019) using the group-averaged tissue response functions for white matter, grey matter and cerebrospinal fluid (Dhollander et al., 2016, 2019). Then, multi-tissue informed log-domain intensity normalization was performed to address bias fields and global intensity variations across participants (Dhollander et al., 2021; Raffelt et al., 2017).

We generated a study-specific unbiased FOD template using all participants’ FOD images and warped the individual FOD images to this template. Next, a whole brain fixel analysis mask was defined by segmenting the fixels from the population FOD template. Similarly, fixels were segmented from the individual subject FOD images in template space. Correspondence was established between each fixel in the fixel analysis mask and a matching fixel in each individual subject FOD image in template space.

The combined measure of FDC was calculated as the product of FD and FC. To this end, FD was computed as the integral of the FOD lobe corresponding to each fixel and FC was computed based on the warps that were generated during registration to the FOD group template (Raffelt et al., 2017).

Finally, whole brain probabilistic tractography was performed on the population template, generating 20 million streamlines and subsequently filtered down to 2 million using spherical-deconvolution informed filtering of tractograms to reduce seeding biases. These tractograms were used for connectivity-based smoothing and statistical inference.

### Segmentation of the Tracts of Interest

The segmentation of the tracts of interest was performed on the group-specific template using the automated Fun With Tracts workflow (Radwan et al., 2022). For this, a group-averaged T1-weighted image was created using MRtrix3 (Tournier et al., 2019). Inclusion and exclusion volumes of interest were automatically generated based on the output of FreeSurfer (Fischl, 2012) and MultiScaleBrainParcellator (Tourbier et al., 2019). Next, bundle-specific tractography was performed using the inclusion and exclusion volumes of interest, followed by streamline filtering and smoothing (Radwan et al., 2022). Finally, tract fixel density maps were created, which were then binarized to create fixel masks of the tracts. Four tracts of interest were chosen, all of which are believed to be part of the structural language connectome and have been implicated in dyslexia by previous studies (see Figure 1A). Two tracts are considered part of the dorsal stream: (1) segment II of the superior longitudinal fasciculus (SLF II), which connects the anterior intraparietal sulcus and angular gyrus with posterior parts of the superior and middle frontal gyri, and (2) the AF, which is made up of three bundles: a long segment connecting the middle and superior temporal gyri with the ventral precentral and inferior and middle frontal gyri, an anterior short bundle linking the supramarginal and superior temporal gyri with the precentral gyrus, and a posterior short bundle bridging the posterior middle temporal gyrus with the angular gyrus. We chose to focus on the second segment of the SLF because Zhao et al. (2016) found alterations of this tract in dyslexia. The two remaining tracts of interest belong to the ventral stream: (1) the IFOF, whose fibers originate in the lingual, posterior fusiform, cuneus and polar occipital cortex, and terminate in the inferior frontal gyrus, medial fronto-orbital region and frontal pole, and (2) the ILF, which extends from occipital and temporal-occipital regions to the anterior temporal pole.

### Statistical Analyses

We conducted analyses across three tiers comparing white matter microstructure between adults with and without dyslexia (see Figure 1B): whole-brain fixel-wise, tract-specific fixel-wise, and tract-specific mean FDC. To account for between-participant variability in DWI quality, the neighboring DWI correlation (NDC) was included as a covariate in all group comparisons. This measure quantifies the correlation between signal intensities of neighboring volumes in q-space, with lower values (<0.4) indicating potential motion artifacts or poor data quality (Yeh et al., 2019). NDC was calculated after image preprocessing using Dipy (Version 1.11.0; Garyfallidis et al., 2014). All group comparisons also included the total intracranial volume as a covariate.

Fixel-based analysis was run on a whole-brain level according to the default settings within MRtrix3 (Tournier et al., 2019) with FDC as outcome variable. Non-parametric permutation testing (with 5,000 permutations) was performed using connectivity-based fixel enhancement (Raffelt et al., 2015), and family-wise error (FWE) corrections were applied to control for false positives. Next, eight tract-specific fixel-based analyses were conducted (four tracts, two hemispheres). These were performed in the same way as the whole-brain analysis, but were confined to the tract-specific fixels using fixel masks of the tracts. Finally, we compared mean FDC values of the tracts of interest using eight one-way analysis of covariance (ANCOVA). In addition, Bayes factors (BFs) were calculated comparing the full ANCOVA model with a null model that only included the covariates. Corrections for multiple comparisons were applied using false-discovery rate (FDR) to adjust the *p* values of the ANCOVAs.

To examine the relationship between white matter microstructure and reading in adults with dyslexia, we ran a series of linear regressions with word reading test performance as dependent variable and FDC, intracranial volume, and NDC as independent variables. These analyses were again performed at three levels of analysis: (1) on a whole-brain fixel level, (2) on a fixel level within the tracts of interest using the FBA framework with permutation testing and FWE corrections, and (3) by correlating the mean FDC values of the tracts with reading performance, applying an FDR adjustment to the effect of FDC to correct for multiple comparisons.

Sensitivity analyses were performed to determine the minimal detectable effect sizes for group differences and correlations, given a power of 0.8, a significance threshold alpha = 0.05, and our sample size, using the pwr package in R.

## RESULTS

### Language Proficiency

**Figure 2.**
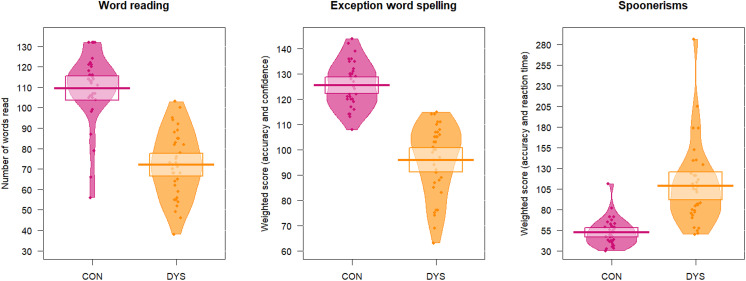
Language proficiency in the control and dyslexia groups. Spoonerisms: lower score equals a better performance. Spelling tests: value for the control group are shown for *n* = 33 as one control participant did not complete this test. Con = Controls, Dys = dyslexics.

As expected, the dyslexic group demonstrated significantly poorer performance on the word reading test, the spelling test and the phonological awareness test (spoonerisms) compared to controls (see Table 1 and Figure 2). This confirms the ongoing nature of the dyslexia related difficulties in our participants with a formal dyslexia diagnosis.

**Table 1.** Demographics

	Control *M* (*SD*)	Dyslexia *M* (*SD*)	*p* value	Effect size
Sex (male/female)	13/21	14/21	1	−0.099
Age (yr)	23.38 (5.10)	23.40 (5.33)	0.989	−0.003
Education (yr)	14.68 (1.89)	13.83 (2.01)	0.075	0.435
ICV (mm^3^)	1567714 (193881)	1561576 (204143)	0.899	0.031
Word reading test	109.47 (17.06)	72.14 (16.07)	<0.001	2.253
Spelling test	125.55 (8.99)	95.97 (13.96)	<0.001	2.504
Spoonerism test	52.12 (16.39)	108.61 (48.46)	<0.001	−1.552

*Note*. Spelling tests: value for the control group are shown for *n* = 33 as one control participant did not complete this test. Effect sizes are Cohen’s *h* for sex and Cohen’s *d* for the other demographics, and *p* values are for comparisons between the control and dyslexia groups (two-proportions z test for sex, two-sided two-sample *t* test for the other demographics). Spoonerisms: lower score equals a better performance. *M* = Mean, *SD* = standard deviation, ICV = intracranial volume.

### Neighboring DWI Correlation

The NDC was calculated as a measure of image quality. The minimum NDC in our sample was 0.77, well above the threshold for poor quality (<0.40; Yeh et al., 2019). While the control group showed a trend towards higher NDC (*M* = 0.824, *SD* = 0.017) compared to the dyslexia group (*M* = 0.811, *SD* = 0.035), this difference did not reach statistical significance according to a two-tailed, two-samples *t* test, *t*(47.06) = 1.88, *p* = 0.066, Cohen’s *d* = 0.45.

### Group Comparisons of White Matter

**Figure 3.**
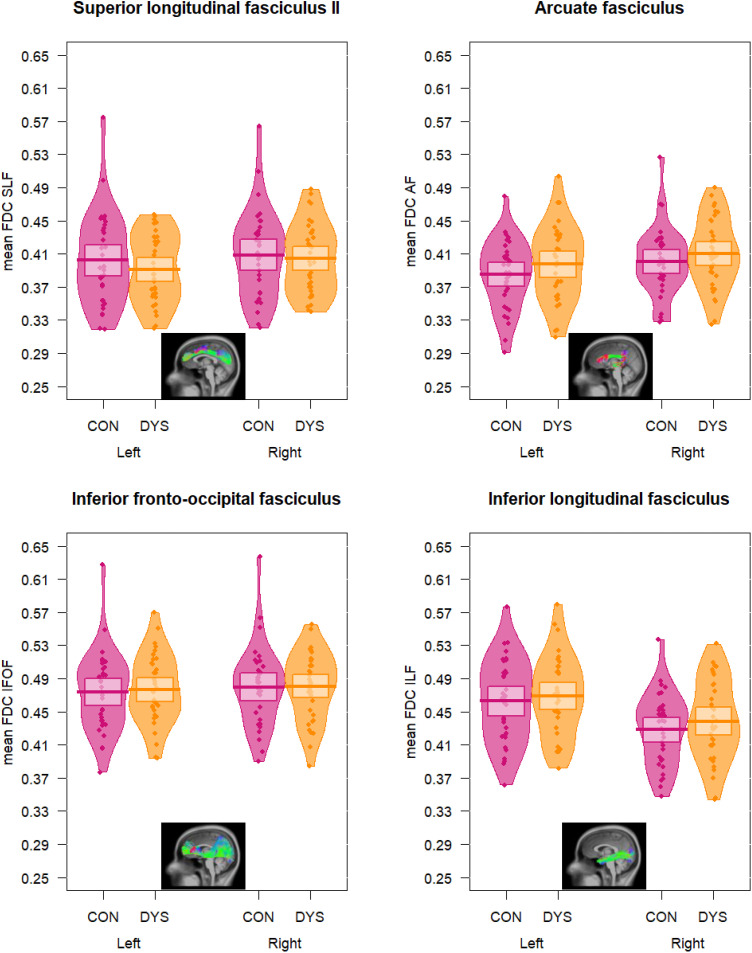
Mean fiber density and cross-section (FDC) values in the tracts of interest. SLF = superior longitudinal fasciculus, AF = arcuate fasciculus, IFOF = fronto-occipital fasciculus, ILF = inferior longitudinal fasciculus. Con = Controls, Dys = dyslexics.

There were no significant group differences in FDC across the three levels of analysis: no significant differences in the whole-brain FBA, nor in the tract-specific FBAs, nor in the mean FDC values of the tracts. Partial eta squared effect sizes for the group effects in the mean FDC values of the tracts of interest ranged from 0.001 to 0.03, which is equivalent to Cohen’s *d* values between 0.06 and 0.35. Bayesian analysis revealed evidence in favor of the null model (intracranial volume as only predictor of mean FDC) over the full model that additionally included the dyslexia versus control group variable, with BFs indicating anecdotal (0.33 < BF < 1: left SLF II, bilateral AF, right ILF) or moderate evidence (0.10 < BF < 0.33: Right SLF II, bilateral IFOF, left ILF). See Figure 3 for descriptive plots of mean FDC values of the tracts of interest in each group and Table 2 for the output of the ANCOVAs.

**Table 2.** ANCOVA results of the tract-specific group comparisons

Tract	Effect	*F*(1, 65)	*p* uncor.	*η* ^2^	*d*	BF
Left SLF II	Group	1.32	0.255	0.020	0.29	0.441
	eTIV	22.16	<0.001	0.25		
	NDC	0.76	0.387	0.01		

Right SLF II	Group	0.16	0.688	<0.01	0.11	0.274
	eTIV	22.55	<0.001	0.26		
	NDC	0.24	0.627	<0.01		

Left AF	Group	1.96	0.167	0.03	0.35	0.582
	eTIV	24.51	<0.001	0.27		
	NDC	0.027	0.871	<0.01		

Right AF	Group	1.33	0.253	0.02	0.29	0.443
	eTIV	23.89	<0.001	0.27		
	NDC	<0.01	0.993	<0.01		

Left IFOF	Group	0.17	0.678	<0.01	0.11	0.274
	eTIV	26.25	<0.001	0.29		
	NDC	0.06	0.812	<0.01		

Right IFOF	Group	0.16	0.693	<0.01	0.08	0.264
	eTIV	30.54	<0.001	0.32		
	NDC	0.975	0.327	0.02		

Left ILF	Group	0.08	0.777	<0.01	0.06	0.260
	eTIV	24.86	<0.001	0.28		
	NDC	2.69	0.106	0.04		

Right ILF	Group	0.76	0.388	0.01	0.21	0.352
	eTIV	26.65	<0.001	0.29		
	NDC	1.37	0.245	0.021		

*Note*. *p* uncor. = uncorrected *p* value; *η*^2^ = generalized eta squared; *d* = Cohen’s *d* equivalent of *η*^2^ for the Group effect; BF = Bayes Factor comparing full model with eTIV-only model; eTIV = estimated total intracranial volume; SLF = superior longitudinal fasciculus; AF = arcuate fasciculus; IFOF = fronto-occipital fasciculus; ILF = inferior longitudinal fasciculus.

### Associations Between White Matter FDC and Word Reading Performance

**Figure 4.**
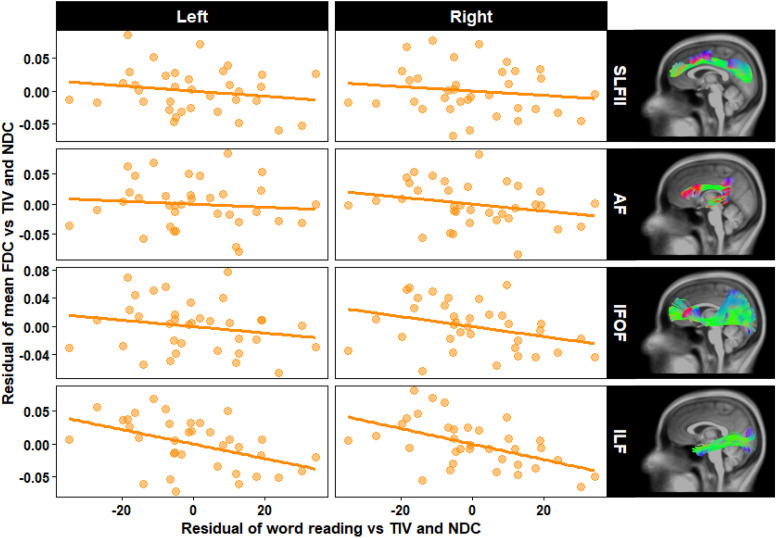
Associations between mean FDC of the tracts-of-interest and reading performance in the dyslexia group. SLF = superior longitudinal fasciculus, AF = arcuate fasciculus, IFOF = fronto-occipital fasciculus, ILF = inferior longitudinal fasciculus. TIV = total intracranial volume. NDC = Neighboring DWI Correlation.

Regression analyses revealed significant negative associations between single-word reading scores and mean FDC values of both the left and right ILF in the dyslexia group (*β* = −0.57, *p*_uncorrected_ = 0.008, *p*_FDR_ = 0.030, and *β* = −0.68, *p*_uncorrected_ = 0.002, *p*_FDR_ = 0.016, respectively). The mean FDC of the other tracts of interest did not correlate significantly with the single word reading scores, with standardized *β* coefficients ranging from −0.12 to −0.41. Similarily, tract-specific fixel-level analyses revealed significant negative associations between reading performance and the FDC in several fixels of the left ILF (16 significant fixels) and right ILF (4 significant fixels). None of the remaining fixel-level regression analyses reached statistical significance (not whole brain nor within the tracts of interest). Table 3 displays the results of the tract-average regression analyses and Figure 4 shows the scatter plots of the residuals of the language test scores and mean FDC values after total intracranial volume and NDC were regressed out.

**Table 3.** Regression table for mean tract FDC values and reading performance in the dyslexia group

Predictor	Statistic	Tracts							
		L AF	R AF	L SLF II	R SLF II	L IFOF	R IFOF	L ILF	R ILF
mean FDC	*β*	−0.119	−0.305	−0.229	−0.169	−0.237	−0.409	−0.565	−0.682
	*p* uncor.	0.573	0.149	0.299	0.417	0.257	0.056	0.008	0.002
	*p* FDR	0.573	0.299	0.398	0.477	0.398	0.150	0.030	0.016

eTIV	*β*	0.189	0.288	0.244	0.208	0.253	0.364	0.430	0.554
	*p* uncor.	0.365	0.168	0.246	0.309	0.227	0.087	0.032	0.009

NDC	*β*	0.025	0.000	−0.016	0.015	0.026	0.021	−0.077	−0.056
	*p* uncor.	0.890	10.000	0.932	0.936	0.886	0.901	0.646	0.724

*R* ^2^		0.028	0.082	0.052	0.038	0.058	0.128	0.223	0.282
*R* ^2^_Adj._		−0.066	−0.007	−0.040	−0.055	−0.033	0.044	0.147	0.212

*Note*. *β* = standardized regression coefficient, *p* uncor. = uncorrected *p* value, *p* FDR = false discovery rate corrected *p* value, L = left hemisphere, R = right hemisphere, AF = arcuate fasciculus, SLF II = superior longitudinal fasciculus , IFOF = fronto-occipital fasciculus, ILF = inferior longitudinal fasciculus, eTIV = estimated total intracranial volume, NDC = neighboring DWI correlation.

### Sensitivity Power Analysis

Sensitivity power analyses revealed our sample size afforded us a minimal detectable group difference of Cohen’s *d* = 0.684 and a minimal detectable correlation of Pearson’s *r* = 0.453 with power = 0.80 and significance threshold alpha = 0.05.

## DISCUSSION

This study examined fiber-specific properties of white matter in dyslexic adults compared with controls and their association with word reading proficiency across three different levels of analysis granularity. While addressing methodological limitations of prior studies, and despite the dyslexic group demonstrating expected deficits in reading, spelling, and phonological awareness, none of our analyses revealed significant differences in white matter structure between dyslexic and non-dyslexic participants. On the contrary, Bayesian analyses suggested anecdotical to moderate evidence supporting the absence of group effects on the mean FDC of all four preselected reading-associated tracts of interest (SLF II, AF, IFOF, and ILF) in our sample. However, we did observe a significant correlation between poorer word reading performance and higher FDC in the bilateral ILF among dyslexic participants only.

The null results of the group comparisons in the current study align with the conclusions drawn by Moreau, Stonyer, et al. (2018) in their voxel-based meta-analysis and Ramus et al. (2018) in their systematic review, both of which noted that white matter differences between dyslexics and neurotypical readers replicate poorly across studies. Our findings also parallel those of a recent large-scale FBA study by Meisler and Gabrieli (2022), who examined the relationship between reading and white matter in a large open dataset of 983 children. Although they found positive associations between FDC and reading skills throughout the brain, especially in left temporoparietal and cerebellar white matter, group comparisons between typical readers and participants with reading difficulties did not reveal significant differences. The empirical study by Moreau, Wilson, et al. (2018) similarly reported unaltered FA within the bilateral SLF and corona radiata, which they selected as tracts of interest, in adults with dyslexia. In line with our findings, their Bayesian analysis also provided weak to moderate evidence supporting the absence of group differences. However, our results contrast with several preceeding studies that did report statistically significant group effects (Banfi et al., 2019; Farah et al., 2022; Vanderauwera et al., 2017; Vandermosten, Boets, Poelmans, et al., 2012; Vandermosten, Boets, Wouters, et al., 2012; Zhao et al., 2016). Studies that reported effect sizes for their significant group differences or provided sufficient information for their calculation (summarized in Supplementary Materials 1, available at https://doi.org/10.1162/NOL.a.17) suggest moderate to strong effects, with Cohen’s *d* values ranging from 0.541 to 0.919 (Banfi et al., 2019; Vandermosten, Boets, Poelmans, et al., 2012; Zhao et al., 2016). These reported effects exceed the maximum effect size observed in our study (Cohen’s *d* = 0.35), and most—but not all—were detectable given power = 0.80 according to our sensitivity analyses. While significant correlations between DWI parameters and reading or reading-adjacent performance reported by earlier dyslexia research can reach similar moderate to high values, the exceptionally well-powered study by Meisler and Gabrieli (2022) observed significant correlations as low as 0.167. The current study lacked sufficient statistical power to detect such subtle associations.

To explain the absence of group differences in our study we discuss three potential explanations. The most direct interpretations of our results are that white matter structure might be normalized by adulthood or is unaltered in adult dyslexia, with the latter suggesting that its neurobiological correlates may instead lie in other aspects of brain structure or function, such as regional hypo- or hyperactivity (Martin et al., 2016; Müller-Axt et al., 2025; Richlan et al., 2009; Yan et al., 2021), abberant functional connectivity (Turker et al., 2023), or cerebral laterality (Packheiser et al., 2023; Zhao et al., 2016). Alternatively, white matter structure in dyslexics might differ from neurotypical readers, but without a systematic pattern across individuals. The pronounced etiological heterogeneity of dyslexia, together with interindividual differences in developmental brain-environmental dynamics, could differentially affect white matter development (Banfi et al., 2019; Wolf et al., 2024). This would explain the mixed findings in group differences between studies and the inconsistencies in the involved white matter tracts and direction of effects (e.g., increased or decreased FA in dyslexia) when group effects are present (Banfi et al., 2019; Farah et al., 2022; Sihvonen et al., 2021; Slaby et al., 2023; Vandermosten, Boets, Poelmans, et al., 2012; Zhao et al., 2016). A final explanation is that variations in research design and analytical approaches between past DWI studies and ours could account for the discrepancy in the results. In what follows we highlight three major methodological decisions that set our study apart from most of the current literature.

First, in contrast to many earlier studies that used tensor-based diffusion models, we employed more advanced fixel-based analyses, which yield more biologically plausible indices of white matter properties. Since metrics derived from DTI do not neatly map on those obtained by more sophisticated diffusion models, statistical tests run on these metrics can produce divergent results. One example of how the selection of the diffusion model can influence the outcome of statistical testing is provided by the study of Zhao et al. (2016). While they observed a significant reduction in the FA of the left AF in dyslexics when using a DTI model, this effect disappeared when the same data were analyzed with a more advanced constrained spherical deconvolution algorithm. Nevertheless, white matter alterations in dyslexia have also been reported by studies using contemporary non-tensor methods (Banfi et al., 2019; Zhao et al., 2016), suggesting that differences in diffusion models are likely not the sole explanation for our unexpected null findings.

Second, unlike the bulk of the existing literature, which examined reading difficulties in children, we recruited adult participants. White matter abnormalities related to dyslexia could be more consistent or pronounced in children, but diminish or grow more idiosyncratic by adulthood, for example, due to individuals adopting different compensation strategies or experiencing varying effectiveness of, or access to, reading interventions. As such, white matter effects might only exist or be more detectable in children. However, age is unlikely to be the only reason for our results deviating from the literature, as white matter alterations have been observed in adult dyslexics (Sihvonen et al., 2021; Vandermosten, Boets, Poelmans, et al., 2012), although one study reported null results (Moreau, Wilson, et al., 2018). Likewise, a previous DTI study on children with dyslexia found no group differences either (Koerte et al., 2016). In addition, it remains unknown whether age matters. No DWI study has investigated whether white matter abnormalities associated with dyslexia are more pronounced in childhood and attenuate by adulthood. It would be valuable for future research to explicitly address possible age effects by comparing pediatric and adult samples cross-sectionally or tracking white matter development longitudinally from childhood to adulthood.

Finally, with 35 dyslexics and 34 controls, the current study has gathered a participant cohort that is notably larger than most past DWI research on dyslexia. The first wave of studies usually recruited 10 or fewer volunteers per subgroup, while contemporary studies generally include around 20 participants per subgroup and seldom exceed 30.

Inconsistencies in the literature may be due to limited sample sizes combined with publication bias (Ramus et al., 2018). Small-scale, underpowered studies by themselves are no more likely to produce false positive results but do have lower chances to detect true effects. However, due to the tendency to publish positive results only while shelving null findings, statistically significant effects are less likely to be genuine when reported by underpowered studies (Button et al., 2013). This issue, aggravated by a lack of proper multiple testing correction in some studies, poses a risk that many group differences in the current literature are, in fact, false positives (Ramus et al., 2018).

Addressing this problem requires additional research using larger participant samples and efforts to pool data from individual studies, for example, by uploading raw data to public repositories. Data integration could be a particularly important strategy as it offers the additional benefit of improving methodological homogeneity by allowing different datasets to be analyzed with the same pipeline. Lastly, to maintain balance in the literature, for example, in terms of meta-analytical integration, researchers should not shy away from publishing null results. Adopting Bayesian inferences can further enhance the informativeness of studies reporting null results by quantifying the strength of the evidence against white matter differences in dyslexia, as was done in the current study.

The only effects in the current study that remained statistically significant after correcting for multiple testing were the negative associations between word reading proficiency and the FDC of the bilateral ILF within the dyslexia group. The fact that this effect is clear when FDC is averaged across the tract, but is significant in only a handful of fixels in the tract-specific fixel-level analysis, could reflect the different interpretations of tract-averaged and fixel-level approaches, and highlights the advantage of combining both in a single study. As discussed in more detail by Meisler and Gabrieli (2022), fixel-level analyses can only capture highly localized and consistent effects that are common among participants within specific fixels. In contrast, the tract-averaging approach trades in this spatial specificity for the ability to detect effects even when the precise locations within the tract that drive the effect vary across individuals. Our findings thus suggest that a general increase of FDC within the left and right ILF, irrespective of its exact location within the tract, could likely pair with poorer word reading in dyslexia. Although Banfi et al. (2019) similarly reported a negative correlation between literacy performance and the FA of the right ILF, but not the left, in their sample of children with dyslexia, our finding is still unexpected since higher FDC values indicate superior information transfer capabilities of a white matter tract (Raffelt et al., 2017). While it cannot be ruled out definitively that the associations we observed may be false positives, the fact that they exceed the minimally detectable effect size indicated by our sensitivity analysis and survive FDR correction provides some support against this interpretation. An alternative, admittingly post hoc, explanation for this finding is that it may flag a maladaptive (over)reliance on the ILF that might have hindered the development of more appropriate forms of neural compensation.

## ACKNOWLEDGMENTS

We would like to acknowledge Thijs Dhollander for his help with the dMRI sequence installation and his guidance during the fixel-based analyses. We would also like to thank all participants who took part in this study. Their willingness to share their time has been invaluable in helping us advance our understanding of dyslexia.

## FUNDING INFORMATION

Helena Verhelst, Fonds Wetenschappelijk Onderzoek (https://dx.doi.org/10.13039/501100003130), Award ID: 1217621N.

## AUTHOR CONTRIBUTIONS

**Helena Verhelst**: Conceptualization: Lead; Data curation: Lead; Formal analysis: Equal; Funding acquisition: Lead; Investigation: Lead; Methodology: Equal; Visualization: Supporting; Writing – original draft: Equal; Writing – review & editing: Supporting. **Robin Gerrits**: Formal analysis: Equal; Methodology: Supporting; Visualization: Lead; Writing – original draft: Equal; Writing – review & editing: Lead. **Emma Karlsson**: Investigation: Supporting; Methodology: Supporting; Writing – review & editing: Supporting.

## DATA AND CODE AVAILABILITY

Anonymized MRI data are freely available on OpenNeuro at https://openneuro.org/datasets/ds005577/versions/1.0.0. The processed data and processing code have been made publicly accessible on Open Science Framework at https://osf.io/znq69/.

## Supplementary Material



## TECHNICAL TERMS

Activation likelihood estimation: A statistical method used to identify brain regions consistently activated across multiple neuroimaging studies.
Fixel-based analysis: A neuroimaging technique analyzing specific fiber segments in brain white matter to assess microscopic structural differences.
Myelination: The process of forming protective, insulating layers around nerve fibers, improving signal transmission in the brain and nervous system.
