# Supplementary material for: No Support for White Matter Alterations in Adults With Dyslexia: A Fixel-Based Diffusion MRI Study
Source: Neurobiol Lang (Camb). 2025 Sep 24;6:NOL.a.17. doi: 10.1162/NOL.a.17 (PMC12534028; doi:10.1162/NOL.a.17)
Supplement: Supplementary file 1 [file nol-6-1-17-s001.pdf]

Supplementary Materials 1

To contextualize the effect size observed in the current study, we retrieved or calculated effect sizes from (statistically significant) group comparisons of diffusion-weighted imaging (DWI) parameters between individuals with and without dyslexia, as reported in previous studies. These values are tabulated below. It is important to note that this is not a systematic review, and our analysis may therefore not be exhaustive. Furthermore, due to limitations in the available data, our analysis was restricted to averaged values within specific white matter tracts or their segments, and does not include voxel- or fixel-wise results . As such, the reported effect sizes should be considered indicative rather than fully representative of the broader body of literature.

Study	Method	Source	Tract	DYS	CO	Cohen's d
Vandermosten 2012	Mean FA in tract-of-interest	Calculated from Table 2	L AF	20	20	**.654**
			L AF direct	20	20	**.528**
			L AF ant.	20	20	-.300
			L AF. post	20	20	.415
			R AF	20	20	-.156
			R AF direct	9	12	.584
			R AF ant.	20	20	.000
			R AF. post	20	20	.222
			L IFOF	20	20	-.039
Zhao 2016	HMAO in tract-of-interest	Reported in Results	L SLF	32	32	< .063
			R SLF	32	32	**-.732**
			L IFOF	32	32	.155
			R IFOF	32	32	.573
Banfi 2019	FA diffusion profiles in tract-of-interest	Calculated from Supp. Table 1*	L AF	27	27	.000 to .022
			R SLF	27	27	**-.600 to -.640**
			L ILF	27	27	**-.724 to -.919**
			R ILF	27	27	**-.541 to -.611**

DYS = Number of participants of dyslexia; CO = Number of controls; Cohen’s d values in bold indicate statistical significant results; HMAO = Hindrance-modulated oriented anisotropy.* For *Banfi, et al., (2019)*, a range of Cohen’s d’s values is reported per tract, as FA was quantified along 100 sections within each tract.
